# Toxic Effects of Urethane Dimethacrylate on Macrophages Through Caspase Activation, Mitochondrial Dysfunction, and Reactive Oxygen Species Generation

**DOI:** 10.3390/polym12061398

**Published:** 2020-06-22

**Authors:** Chih-Yang Chang, Chen-Yu Chiang, Yun-Wei Chiang, Min-Wei Lee, Chien-Ying Lee, Hung-Yi Chen, Hui-Wen Lin, Yu-Hsiang Kuan

**Affiliations:** 1 Division of Pediatric Surgery, Department of Pediatrics, Ditmanson Medical Foundation Chia-Yi Christian Hospital, 600 Chia-Yi City, Taiwan; 00880@cych.org.tw; 2 Department of Veterinary Medicine, National Chung-Hsing University, 402 Taichung, Taiwan; online70222@gmail.com; 3 Department of Life Sciences, National Chung-Hsing University, 402 Taichung, Taiwan; apple3793@gmail.com; 4 A Graduate Institute of Microbiology and Public Health, National Chung Hsing University, 402 Taichung, Taiwan; cat852654@gmail.com; 5 Department of Pharmacology, School of Medicine, Chung Shan Medical University, 402 Taichung, Taiwan; cshd015@csmu.edu.tw; 6 Department of Pharmacy, Chung Shan Medical University Hospital, 402 Taichung, Taiwan; 7 School of Pharmacy, China Medical University, 404 Taichung, Taiwan; hungyi@mail.cmu.edu.tw; 8 Department of Optometry, Asia University, 413 Taichung, Taiwan; 9 Genetics Center, Department of Medical Research, China Medical University Hospital, and School of Chinese Medicine, China Medical University, 404 Taichung, Taiwan

**Keywords:** UDMA, macrophage, genotoxicity, apoptosis, ROS generation, caspase activation

## Abstract

Urethane dimethacrylate (UDMA) is a dimethacrylate-based resin monomer that can react with other related monomers and inorganic particles, causing hydrophobic polymerization through cross-linking upon light activation. UDMA polymers are commonly used for the reconstruction and reinforcement of teeth and bones. UDMA can become unbound and be released from light-cured polymer resins. Thus far, no evidence exists on the toxic effects of UDMA and its related working mechanisms for macrophages. Therefore, in the present study, we investigated the cytotoxicity, mode of cell death, DNA damage, caspase activities, mitochondrial dysfunction, and reactive oxygen species (ROS) generation in RAW264.7 macrophages treated with UDMA using the lactate dehydrogenase (LDH) assay kit, Annexin V-FITC and PI assays, micronucleus formation and comet assay, caspase fluorometric assay, JC-1 assay, and 2ʹ,7ʹ-dichlorofluorescin diacetate (DCFH-DA) assay, respectively. Our results show that UDMA induced cytotoxicity; apoptosis and necrosis; genotoxicity, which is also called DNA damage; increased caspase-3, -8, and -9 activities; mitochondrial dysfunction; and intracellular ROS generation in a concentration-dependent manner in RAW264.7 macrophages. Thus, based on the observed inhibited concentration parallel trends, we concluded that UDMA induces toxic effects in macrophages. Furthermore, UDMA-induced intracellular ROS generation, cytotoxicity, and DNA damage were reduced by N-acetyl-L-cysteine.

## 1. Introduction

Urethane dimethacrylate (UDMA) is a dimethacrylate-based resin monomer used in restorative dentistry and the repair of bone tissue [1]. UDMA and related monomers, including bisphenol A glycol dimethacrylate (BisGMA), triethylene glycol dimethacrylate (TEGDMA), and bisphenol A ethoxylated dimethacrylate (BisEMA), with their inorganic particles are activated by light to induce hydrophobic polymerization through cross-linking [2]. Such polymers are used for the reconstruction and reinforcement of teeth and bones. UDMA exists in an unbound state in light-cured polymers. Moreover, UDMA and its related dimethacrylate monomers can be released from the light-cured polymeric resins and can enter the peripheral tissues, oral cavity, and pulp [3]. Unbound UDMA or UDMA leakage would induce harmful reactions in the peripheral environment. UDMA is the main leached component from dental bonding agents or resin composites, causing impaired healing, inflammation, and necrosis in pulp tissue [4]. Toxic effects, including cytotoxicity, genotoxicity, and apoptosis, were induced by UDMA in human pulp cells and Chinese hamster ovary (CHO) cells [5,6,7].

Macrophages, which play a critical role in the immune system, are ubiquitous in many peripheral tissues, including oral tissue. They serve as the first line of defense against invading microbial pathogens and unfamiliar chemicals. Pathogens and chemicals may cause overactivation of macrophages, resulting in an inflammatory response [8] and leading to pulpitis, periodontitis, and oral ulcers [9]. Based on the inflammatory reactions induced by dental bonding agents or resin composites in pulp tissue, we hypothesize that macrophages are affected by UDMA. Therefore, in the present study, we investigated the toxic effects of UDMA, such as DNA damage, cysteine-containing aspartate-specific protease (caspase) activities, and reactive oxygen species (ROS) generation, on a murine macrophage cell line, RAW264.7.

## 2. Materials and Methods 

### 2.1. Materials

Dulbecco’s Modified Eagle Medium (DMEM), fetal bovine serum (FBS), and other cell culture reagents were obtained from Thermo Fisher Scientific (Eugene, OR, USA). UDMA, cytochalasin B, dimethyl sulfoxide (DMSO), 5,5,6,6-tetrachloro-1,1,3,3-tetraethylbenzimidazolylcarbocyanine iodide (JC-1), phosphate buffered saline (PBS), and other chemical reagents were obtained from Sigma-Aldrich (St. Louis, MO, USA). Caspase-3, -8, -9 fluorometric assay kits were purchased from Alexis Biochemicals (Enzo Life Sciences, Plymouth Meeting, PA, USA).

### 2.2. Cell Culture and Cell Treatment

The murine macrophage cell line RAW264.7 (BCRC No.6001) was obtained from the Bioresource Collection and Research Centre of the Food Industry Research and Development Institute (Hsinchu, Taiwan). The cells were cultured in DMEM supplemented with 10% FBS, 100 U/mL penicillin, and 100 μg/mL streptomycin in a humidified incubator at 37 °C with 5% CO_2_ and 95% air. Confluent cells were detached using 0.25% trypsin and 0.05% EDTA for 5 min, and aliquots of the separated cells were subcultured at 1:4 splits every 3 days [10]. After incubation with or without N-acetyl-L-cysteine (NAC) at a concentration of 10 μM for 30 min, the cells were treated with UDMA at various concentrations of 0, 1, 10, and 100 μM for another 24 h for further experiments.

### 2.3. Lactate dehydrogenase (LDH) Release Assay

Cytotoxicity induced by UDMA in RAW264.7 cells was measured by the level of LDH released [11]. RAW264.7 cells were incubated with UDMA at various concentrations for 24 h. The positive control group was incubated with a lysis solution to achieve the maximum level of LDH. The supernatant medium was incubated using the CytoTox 96 Non-radioactive Cytotoxicity Assay Kit (Promega, Madison, WI, USA) according to the manufacturer’s instructions. Absorbance was detected at 490 nm using the Synergy HT multi-mode microplate reader (Biotek, Winooski, VT). The percentage of cytotoxicity was calculated as the ratio of LDH released from the cells treated with UDMA to that from the positive controls.

### 2.4. Apoptosis and Necrosis Assay

The Annexin V-FITC and PI assay kit (BioVision, San Jose, CA, USA) was used for the detection of apoptosis and necrosis as per the methodology used in a previous study. After treatment with UDMA at various concentrations for 24 h, the cells were collected using ice-cold PBS [8]. The cells were incubated with Annexin V-FITC and PI in a binding buffer solution at 37 °C in the dark. The samples were acquired and analyzed on the BD Accuri C6 flow cytometer using C6 software (BD Biosciences, San Jose, CA, USA). Percentages of cells in the stages of living, early apoptosis, late apoptosis, and necrosis were presented as annexin V−/PI−, annexin V+/PI−, annexin V+/PI+, and annexin V−/PI+, respectively.

### 2.5. Micronucleus (MN) Formation Assay

We used the micronucleus (MN) formation assay to assess the genotoxic effects (i.e., DNA damage) caused by UDMA, as previously described [12]. Briefly, the cells were treated with UDMA at various concentrations, 3 mg/mL cytochalasin B, and 0.17 μg/mL mitomycin C for 24 h. Later, the cells were washed, resuspended in 75 mM KCl, and then fixed in a mixture of methanol/acetic acid. They were stained using 3% Giemsa solution. MN was analyzed microscopically in 1000 binucleated cells per concentration.

### 2.6. Single-Cell Gel Electrophoresis (Comet) Assay

To assess the DNA damage caused by UDMA, the comet assay was used, as outlined in a previous study [12]. After treatment, the cells were mixed with low melting agarose and mounted on microscope slides precoated with normal melting agarose and then covered using a coverslip on ice. After the coverslip was removed, the microscope slides were immediately submerged in a lysis solution containing 2.5 M NaCl, 100 mM ethylenediaminetetraacetic acid (EDTA), 10 mM Tris pH 10, 1% Triton X-100, 200 mM NaOH, 34.1 mM N-Lauroyl-Sarcosine, and 10% DMSO at 4 °C for 1 h in the dark. They were then washed and placed in a horizontal electrophoresis tank filled with electrophoresis buffer for 40 min at 4 °C for DNA unwinding and denaturation. The microscope slides were then incubated using a neutralization buffer (0.4 M Tris-HCl, pH 7.5) and stained using ethidium bromide. The results were analyzed using the Comet v.5.5 system (Kinetic Imaging Ltd., Liverpool, UK). To quantify DNA damage, we evaluated the following comet parameters: percentage of DNA in the tail (%DNA in tail; relative fluorescence intensity of tail) and tail length (distance from center of the head to the end of the tail).

### 2.7. Assays to Measure Caspase-3, -8, and -9 Activities

To assess the activation of caspase-3, -8, and -9 induced by UDMA, we used a previously described method [12]. After UDMA treatment, the level of proteins extracted from cells using a lysis buffer for 1 h at 4 °C was measured using the Bradford assay. Equal amounts of lysate protein were incubated with DEVD-AFC, IETD-AFC, and LEHD-AFC, which are fluorogenic substrates of caspase-3, -8, and -9, respectively. After a 2-h incubation at 37 °C, the fluorescence intensity was detected using the microplate reader at excitation and emission wavelengths of 400 and 505 nm.

### 2.8. Mitochondrial Membrane Potential Assay

To assess mitochondrial disruption, the mitochondrial membrane potential (MMP) was detected using JC-1, as described in previous research [10]. The cells were harvested after treatment, and they were incubated with 10 mg/mL JC-1 for 30 min at 37 °C in the dark. Intracellular JC-1 fluorescence was acquired and analyzed on the BD Accuri C6 flow cytometer using C6 software.

### 2.9. Measurement of Intracellular ROS Level

The level of intracellular ROS induced by UDMA was detected using 2ʹ,7ʹ-dichlorofluorescin diacetate (DCFH-DA), as described in previous research [12]. The cells were harvested after treatment, and the cells were incubated with 5 μM DCFH-DA for 30 min at 37 °C in the dark. Intracellular DCF fluorescence was assayed using the microplate reader (Biotek, Winooski, VT, USA) at excitation and emission wavelengths of 488 and 525 nm, respectively.

### 2.10. Statistical Analysis

All experiments were performed in triplicate, and the results were analyzed using SPSS software (IBM, New York, NY, USA). The results are expressed as mean ± standard deviation (SD). The significance of differences was calculated using one-way ANOVA, followed by the Bonferroni’s test for multigroup comparison. A *P* value of less than 0.05 was considered statistically significant.

## 3. Results

### 3.1. Effects of UDMA on Cytotoxicity of RAW264.7 Cells

**Figure 1:**
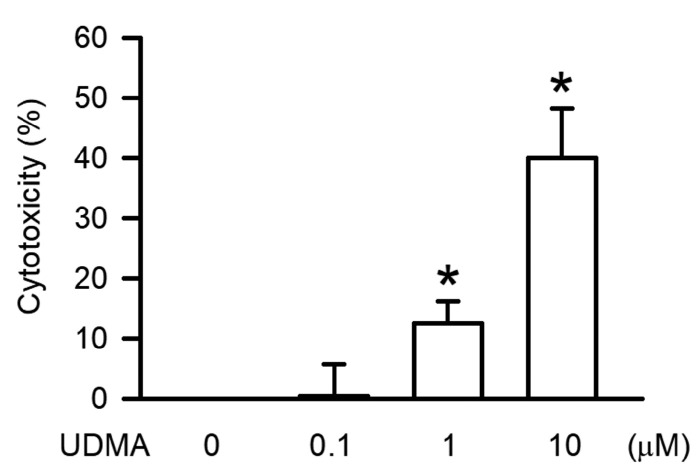
Urethane dimethacrylate (UDMA) induced cytotoxicity in RAW264.7 macrophages. The cells were incubated with UDMA at concentrations of 0, 1, 10, and 100 μM for 24 h at 37 °C. Cytotoxicity was measured using the LDH assay. Data are expressed as mean ± SD (n = 5). * *P* < 0.05 is considered significant compared with the control group.

To determine the cytotoxic effect induced by UDMA in macrophages, the RAW264.7 cells were incubated in UDMA at the indicated concentration for 24 h. The cytotoxicity of RAW264.7 cells treated with UDMA at various concentrations of 0, 1, 10, and 100 μM for 24 h was measured using the LDH release assay (Figure 1). UDMA induced cytotoxicity in a concentration-dependent manner. Treatment with UDMA at 10 μM induced significant cytotoxicity (*P* < 0.05) in RAW264.7 cells.

### 3.2. Effects of UDMA on Necrosis and Apoptosis of RAW264.7 Cells

**Figure 2:**
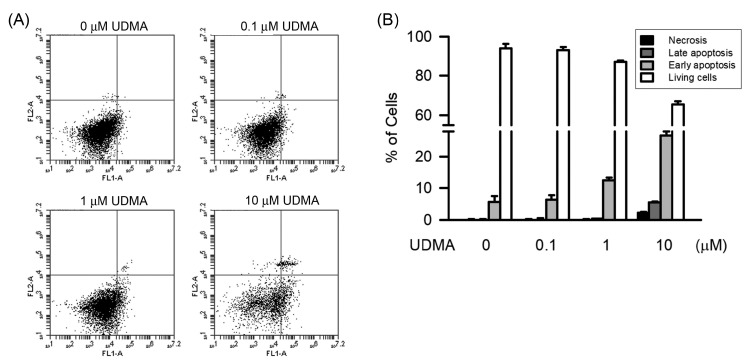
UDMA induced apoptosis and necrosis in RAW264.7 macrophages. The portion of apoptosis and necrosis was measured by Annexin V-FITC and PI assays using flowcytometry. (**A**) Cells were incubated with UDMA at concentrations of 0, 1, 10, and 100 μM for 24 h at 37 °C. The upper left quadrant (Annexin V−/+) is representative of necrosis; the upper right and lower right quadrants (Annexin V+/PI+ and Annexin V+/PI−) are representatives of apoptosis; and the lower left quadrant (Annexin V−/PI−) is representative of living cells. (**B**) Quantitatively, the percentage of necrotic cells, viable cells, and apoptotic cells were calculated and analyzed. Data are expressed as mean ± SD (n = 5). * *P* < 0.05 is considered significant compared with the control group.

To study the role of apoptosis and necrosis, we studied the level of apoptosis and necrosis induced by UDMA in RW264.7 cells. As shown in Figure 2A, apoptosis and necrosis of RAW264.7 cells caused by UDMA were assessed using the annexin V-FITC and PI assay kit and a flow cytometer. After 24-h treatment with UDMA at concentrations of 10 and 100 μM, the cells in early apoptosis significantly increased (*P* < 0.05), from 5.56% to 12.43%–26.73%, quantitatively. The cells in late apoptosis and necrosis also significantly increased (*P* < 0.05) after treatment with 100 μM of UDMA, from 0.17% to 5.47% and 0.2–2.3%, respectively (Figure 2B). These results indicate that UDMA induced early apoptosis at low concentrations and late apoptosis and necrosis at high concentrations.

### 3.3. Effects of UDMA on MN Formation in RAW264.7 Cells

**Figure 3:**
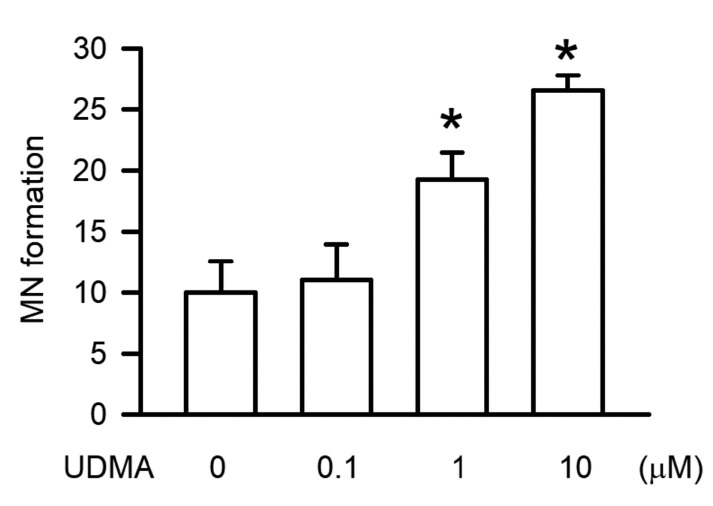
UDMA induced micronucleus (MN) formation in RAW264.7 macrophages. Data are expressed as mean ± SD (n = 5). * *P* < 0.05 is considered significant compared with the control group.

Genotoxicity plays a major role in the regulation of cytotoxicity via apoptosis. As shown in Figure 3, UDMA-induced genotoxic effects were measured using the MN assay. Our results show that UDMA induced MN formation significantly in a concentration-dependent manner, with significant effects beginning at a concentration of 10 μM (*P* < 0.05).

### 3.4. Effects of UDMA on DNA Damage in RAW264.7 Cells

**Figure 4:**
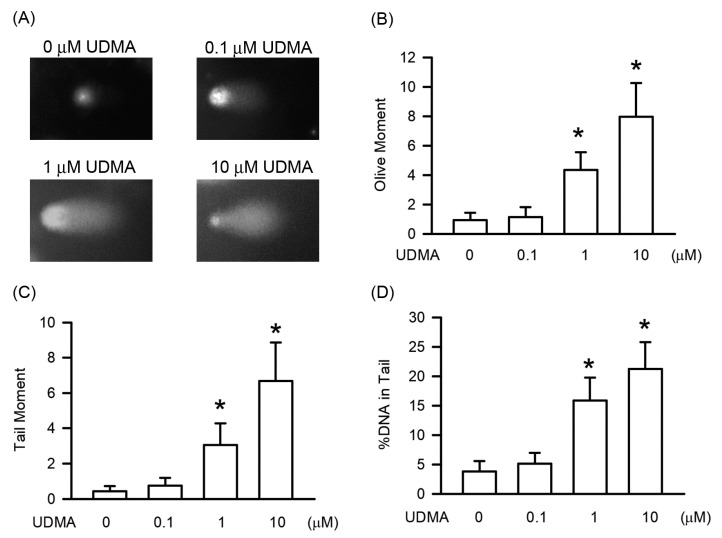
UDMA-induced DNA damage measured using the comet assay in RAW264.7 macrophages. (**A**) Gel electrophoresis of UDMA-treated RAW264.7 cells at concentrations of 0, 1, 10, 100 μM. (**B**) and (**C**) are quantifications of tail moment and (**D**) %DNA in tail, respectively. Results are expressed as mean ± SD. * *P* < 0.05 is considered significant compared with the control group.

DNA damage is the most important of the key features in the genotoxicity. As shown in Figure 4, UDMA-induced DNA damage was detected using the comet assay. Our results show that compared with controls, the tail length (Figure 4B) and %DNA in the tail (Figure 4C) significantly increased in a concentration-dependent manner, with significant effects beginning at a concentration of 10 μM (*P* < 0.05)

### 3.5. Effects of UDMA on Caspase-3, -8, and -9 Activities in RAW264.7 Cells

**Figure 5:**
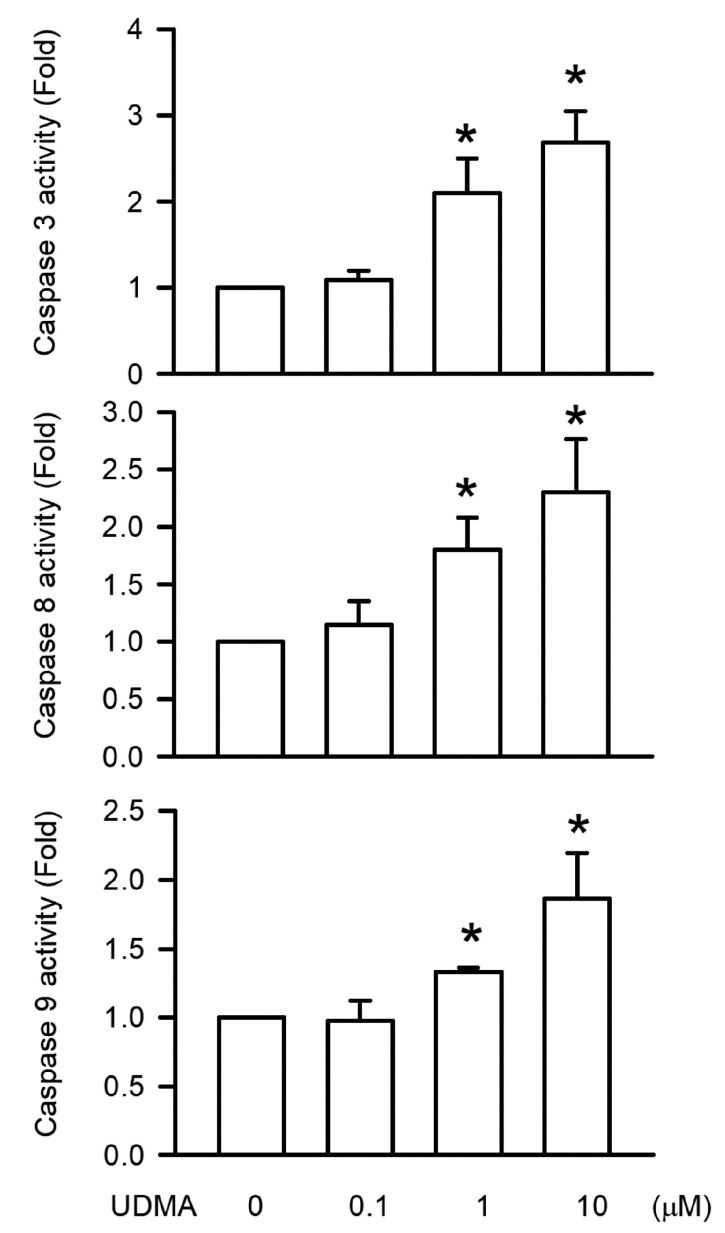
UDMA-induced caspase-3, -8, and -9 activation in RAW264.7 macrophages. Data are expressed as mean ± SD (n = 5). * *P* < 0.05 is considered significant compared with the control group.

Caspase-3, caspase-8, and caspase-9 play important roles in apoptosis and DNA damage in cells. We measured caspase-3, -8, and -9 activities induced by UDMA using caspase fluorometric assay kits. After RAW264.7 cells were treated with UDMA, we observed that caspase-3, -8, and -9 activities were induced by UDMA in a concentration-dependent manner, with significant effects beginning at a concentration of 10 μM (*P* < 0.05, Figure 5).

### 3.6. Effects of UDMA on Mitochondrial Dysfunction in RAW264.7 Cells

**Figure 6:**
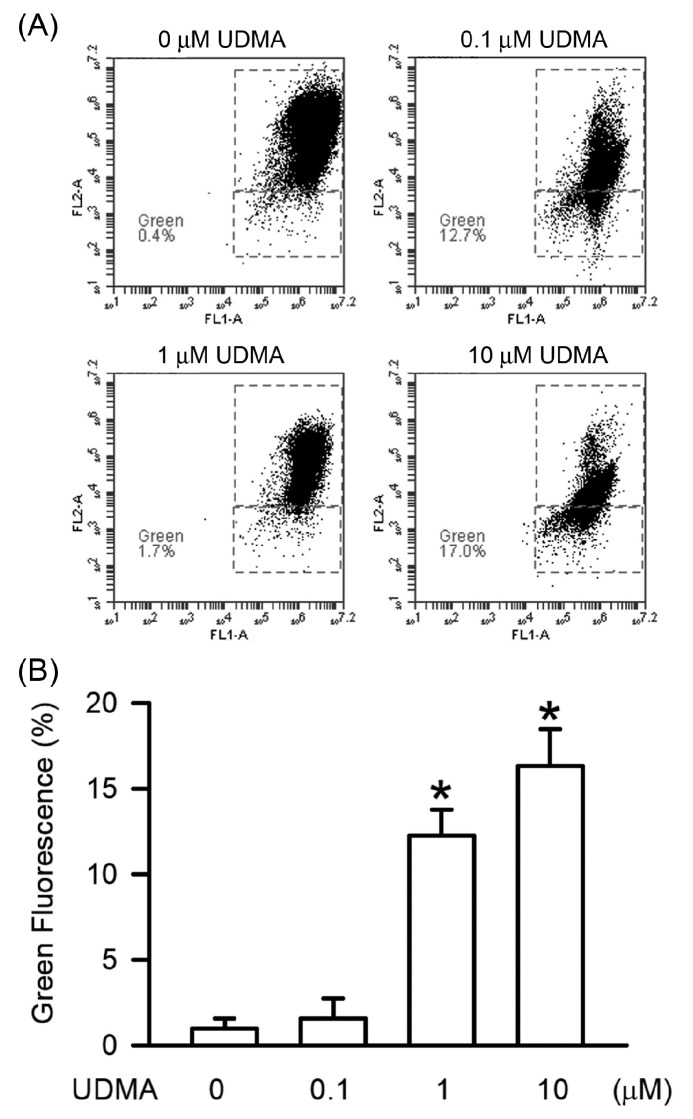
UDMA induced mitochondrial dysfunction in RAW264.7 macrophages. (**A**) Cells were incubated with UDMA at concentrations of 0, 1, 10, and 100 μM for 24 h at 37 °C. The portion of mitochondria dysfunction was measured using the JC1 assay. (**B**) Quantitatively, the percentage of cells with mitochondrial dysfunction were calculated and analyzed. Data are expressed as mean ± SD (n = 5). * *P* < 0.05 is considered significant compared with control group.

Mitochondrial dysfunction is the critical hallmark of apoptosis through caspase activation. As shown in Figure 6, UDMA-induced mitochondrial dysfunction was detected using a fluorescence-based mitochondria-specific voltage-dependent dye, JC-1. As shown in Figure 6A, the green fluorescence of JC1, indicating mitochondrial integrity, was induced by UDMA. Thus, our results showed that UDMA induced mitochondrial dysfunction in a concentration-dependent manner, with significant effects beginning at a concentration of 10 μM (*P* < 0.05, Figure 6B).

### 3.7. Effects of UDMA on Intracellular ROS Generation in RAW264.7 Cells

**Figure 7:**
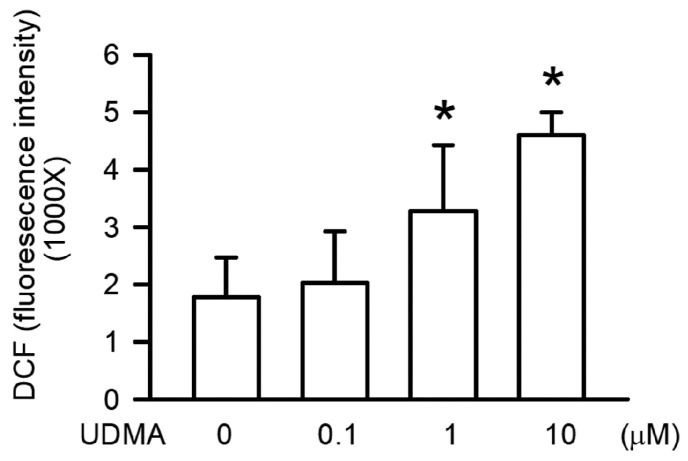
UDMA induced intracellular ROS generation in RAW264.7 macrophages. Data are expressed as mean ± SD (n = 5). * *P* < 0.05 is considered significant compared with control group.

ROS play a major role in genotoxicity and apoptosis. ROS generation in RAW264.7 cells after treatment with UDMA at the indicated concentrations for 24 h was detected using DCFH-DA. As shown in Figure 7, ROS generation was induced by UDMA in a concentration-dependent manner, with a significant increase in the level of ROS observed at a concentration of 10 μM (*P* < 0.05).

### 3.8. Effects of NAC on UDMA-Induced Intracellular ROS Generation, Mitochondrial Dysfunction, and Cytotoxicity in RAW264.7 Cells

**Figure 8:**
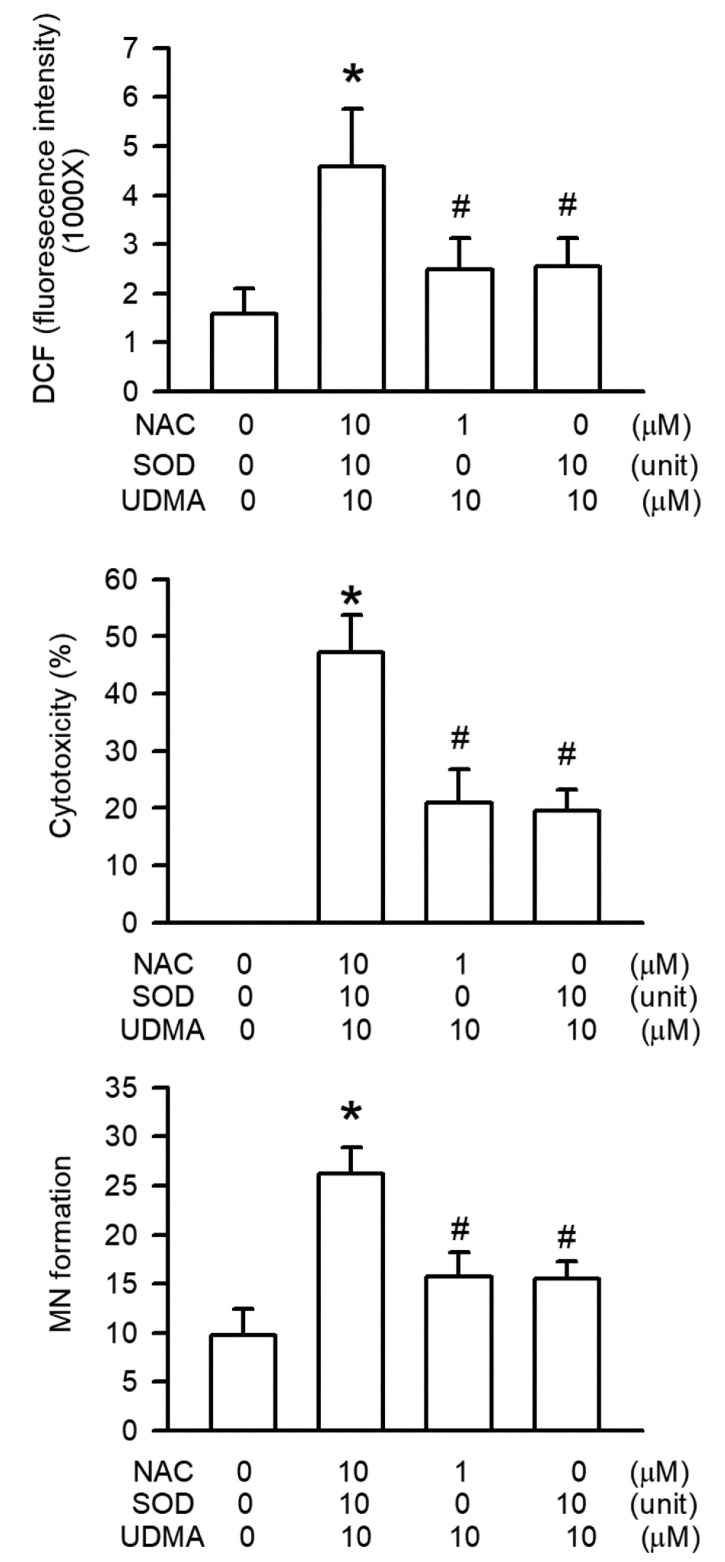
UDMA induced intracellular ROS generation in RAW264.7 macrophages. Data are expressed as mean ± SD (n = 5). * *P* < 0.05 is considered significant compared with control group.

NAC is employed as a cell-permeable ROS scavenger. RAW264.7 cells were pretreated with NAC to evaluate its effects on intracellular ROS generation, cytotoxicity, and mitochondrial dysfunction induced by UDMA. Our results showed that NAC significantly inhibited all three UDMA-induced effects (*P* < 0.05, Figure 8).

## 4. Discussion

Monomers, including BisGMA, TEGDMA, bisphenol A (BPA), and UDMA, are leached from light-cured dimethacrylate dental resins into an aqueous environment at nanomolar ranges [13,14,15]. Our previous studies proposed that cytotoxicity may be induced by BisGMA, TEGDMA, and BPA in macrophages [16,17]. Considerable evidence has shown that cytotoxicity, DNA damage, and apoptosis are induced by UDMA at 1 mM for 1 h in CHO cells [5]. After human dental pulp cells are incubated with UDMA at 0.05 to 0.35 mM for 24 h or 0.1 and 0.3 mM for 48 h, there is a significant reduction in survival rate [6,7]. Human gingival fibroblasts treated with UDMA at 0.1 mM for 24 h also demonstrated a reduction in survival rate [18]. UDMA at the concentration of 0.8 and 1 mM for 24 h induces cytotoxicity in human lymphocytes [19]. However, UDMA-induced cytotoxicity in macrophages has not been demonstrated. Therefore, we investigated this topic, and our findings demonstrate that treatment with 10 μM UDMA for 24 h induced significant cytotoxicity in RAW264.7 macrophages. These results are similar to those of a previous study, where peripheral blood mononuclear cell viability was reduced by 50%–93% upon treatment with UDMA at concentrations of 50–200 μM for 20 h [20]. These findings indicate that UDMA can induce cytotoxicity in human macrophages.

Apoptosis and necrosis are the two major processes of cell death [21,22,23]. Previous studies have implicated UDMA-induced apoptosis in human lymphocytes and CHO cells at 1 mM for 6 h [5,19]. UDMA induced a higher incidence of necrosis than apoptosis in human gingival fibroblasts [18]. After human dental pulp cells were incubated with UDMA at 0.35 mM for 24 h, a significant induction of apoptosis was observed [7]. Our study demonstrated that significant early apoptosis and late apoptosis and necrosis of macrophages are induced by UDMA at concentrations of 10 and 100 μM, respectively. Our results also suggest that the higher the concentration of UDMA, the more severe the cell damage (ranging from apoptosis to necrosis) in RAW264.7 macrophages. A previous study showed, using the comet assay, that UDMA dose-dependently induces cytotoxicity and apoptosis in lymphocytes through DNA damage [5,19]. In the present study, the comet and MN assays revealed DNA damage induced by UDMA, indicating that UDMA induced apoptosis in RAW264.7 macrophages through DNA damage.

The caspases, a family of intracellular cysteine proteases, serve as initiators and executioners in DNA damage and genotoxicity, resulting in apoptosis [24,25]. Caspase-3, the most crucial executioner caspase, promotes DNA damage and is activated by intrinsic and extrinsic pathways initiated by caspase-9 and -8, respectively [24,26]. We demonstrated increased activities of caspase-3, -8, and -9 induced by UDMA in a concentration-dependent manner in RAW264.7 macrophages, which is similar to the finding of UDMA-induced DNA damage and apoptosis. These results imply that apoptosis of macrophages is induced by UDMA through caspase-induced DNA damage.

Mitochondria play critical roles in cellular metabolism, homeostasis, and stress responses by generating ATP for energy and regulating cell death [27]. Mitochondrial dysfunction is usually caused by depolarization and is the early hallmark of toxicity mediated through caspase-induced apoptosis [28]. Our study provides convincing evidence indicating that UDMA dose-dependently caused caspase-induced apoptosis of RAW264.7 cells through mitochondrial dysfunction.

Intracellular ROS generation is a critical feature of mitochondrial dysfunction [29]. A previous study suggested that intracellular ROS generation is induced by UDMA in a concentration-dependent manner [30], and that the precursor of GSH and ROS scavenger, NAC, can prevent ROS generation and cytotoxicity in CHO cells [30]. In the current study, we observed that intracellular ROS generation was induced by UDMA in a concentration-dependent manner in RAW264.7 macrophages, which is similar to the precipitation of UDMA-induced mitochondrial dysfunction. These results thus suggest that mitochondrial dysfunction caused by UDMA occurs through intracellular ROS generation. Moreover, we discovered that NAC reduced UDMA-induced intracellular ROS generation, cytotoxicity, and genotoxicity in RAW264.7 macrophages. Based on these results, we suggest that UDMA-induced intracellular ROS generation from mitochondrial dysfunction resulted in toxic effects on macrophages, including genotoxicity, apoptosis, and cytotoxicity.

In conclusion, UDMA induced cytotoxicity of RAW264.7 macrophages in a concentration-dependent manner, which is associated with early apoptosis at low concentrations in contrast to late apoptosis and necrosis at high concentrations. The mechanisms involved are as follows: (1) DNA damage; (2) activation of caspase-3, -8, and -9; (3) mitochondrial dysfunction; and (4) generation of intracellular ROS. These results suggest that UDMA is a toxic reagent in macrophages. At present, we have enough evidence to support the hypothesis, which is that macrophages are injured by UDMA, which is released from restorative dental composites, and contributes to cytotoxicity and apoptosis via DNA damage, caspases activation, mitochondrial dysfunction, and ROS generation. These findings can be used in the future to develop the new treatment strategies for immune dysfunction related to macrophage damage caused by UDMA.
